# Systems and processes for regulation of investigational medical devices in Uganda

**DOI:** 10.3389/fmedt.2022.1054120

**Published:** 2023-01-23

**Authors:** Charles Norman Mpaata, Brian Matovu, Mercy Takuwa, Noah Kiwanuka, Steff Lewis, John Norrie, Sam Ononge, Sharon Tuck, Maria Wolters, Marc Demulliez, Robert T. Ssekitoleko

**Affiliations:** ^1^ Biomedical Engineering Unit, Department of Physiology, School of Biomedical Sciences College of Health Sciences, Makerere University, Kampala, Uganda; ^2^ Clinical Trials Unit, School of Public Health, College of Health Sciences, Makerere University, Kampala, Uganda; ^3^ Usher Institute, Edinburgh Medical School, University of Edinburgh, Edinburgh, United Kingdom; ^4^ Department of Obstetrics and Gynecology, School of Medicine, College of Health Sciences, Makerere University, Kampala, Uganda; ^5^ Informatics Forum, School of Informatics, College of Science and Engineering, University of Edinburgh, Edinburgh, United Kingdom; ^6^ School of Engineering & Physical Sciences, Heriot-Watt University, Edinburgh, United Kingdom

**Keywords:** medical devices, medical innovation, medical devices regulations, clinical evaluation, medical device standards, regulatory pathway

## Abstract

**Background:**

In many parts of the world, medical devices and the processes of their development are tightly regulated. However, the current regulatory landscape in Uganda like other developing countries is weak and poorly defined, which creates significant barriers to innovation, clinical evaluation, and translation of medical devices.

**Aim:**

To evaluate current knowledge, systems and infrastructure for medical devices regulation and innovation in Uganda.

**Methods:**

A mixed methods study design using the methods triangulation strategy was employed in this study. Data of equal weight were collected sequentially. First, a digital structured questionnaire was sent out to innovators to establish individual knowledge and experience with medical device innovation and regulation. Then, a single focus group discussion involving both medical device innovators and regulators to collect data about the current regulatory practices for medical devices in Uganda. Univariate and bivariate analysis was done for the quantitative data to summarize results in graphs and tables. Qualitative data was analyzed using thematic analysis. Ethical review and approval were obtained from the Makerere University School of Biomedical Sciences, Research and Ethics Committee, and the Uganda National Council for Science and Technology.

**Results:**

A total of 47 innovators responded to the questionnaire. 14 respondents were excluded since they were not medical device innovators. Majority (76%) of individuals had been innovators for more than a year, held a bachelor's degree with a background in Engineering and applied sciences, and worked in an academic research institute. 22 of the 33 medical device innovators had stopped working on their innovations and had stalled at the proof-of-concept stage. Insufficient funding, inadequate technical expertise and confusing regulatory landscape were major challenges to innovation. The two themes that emerged from the discussion were “developing standards for medical devices regulation” and “implementation of regulations in practical processes”. Legal limitations, lengthy processes, and low demand were identified as challenges to developing medical device regulations.

**Conclusions:**

Efforts have been taken by government to create a pathway for medical device innovations to be translated to the market. More work needs to be done to coordinate efforts among stakeholders to build effective medical device regulations in Uganda.

## 1. Introduction

Medical device regulation in Uganda like many other African countries is poorly defined and weak with meagre information available regarding existing regulations, standards, and policies (1, 2). Effective medical device regulations ensure that imported as well as locally developed innovative medical devices are contextually appropriate and adapted to the priority public health conditions, resources, and settings of a particular population (3). As such, rigorous medical device regulations have been developed and published in various parts of the world, especially in high-income countries. For example, the Food and Drugs Administration of the United States of America has a 3-tier medical device classification system, an established premarket notification [510(k)], a premarket approval (PMA) process, and post-market surveillance system for medical devices (4, 5). Similarly, the medical device regulations 2017/745 of the European union provide for a four-class risk classification scheme for medical devices, a Conformité Européenne (CE) marking requirement, and a system of notified bodies to regulate safety and marketing of medical devices (6). Conversely, medical device regulation in developing countries, especially in Africa, is not well developed and is characterized by high degrees of variability and irregularity (7–9).

Whereas many African countries have instituted national regulatory bodies or a government institution to perform some of the functions of medical device regulation (10, 11), these bodies usually lack dedicated committees or personnel to handle medical devices regulation, struggle with lack of funding and usually work in isolation with little or no coordination of efforts (12). Additionally, these organizations lack experience, have no access to technical material and information on medical devices, and have limited oversight (10). Thus, in the absence of specific medical device regulations, inappropriate medical devices have been acquired through donations (13), and many more sub-standard devices have been imported with minimum scientific evidence (14). These devices evoke significant environmental burdens on recipient nations, due to the absence of clear disposal procedures and management or storage of the unusable donated devices (13, 15), which could lead to negative impacts on public health (16). These unsuitable devices present challenges for developing and implementing equipment management strategies and frustrate efforts towards building efficient health systems.

As a result of the poorly defined medical device regulations, innovators in Africa face numerous internal and external challenges in the design and development of much-needed medical devices (17). These include lack of explicit policy support, bureaucracy, organizational rigidity, and deficient financial policies among others (18). Innovators are affected especially by the varyingly inadequate national health research systems and research capacity limitations in their respective countries. This leads to uncertainty among innovators and regulators, unnecessary project delays, inhibit progress to the market and ultimately delay improvements in clinical outcomes. The inconsistencies in recognition of and access to information and tools for medical device regulation also limit the ability of locally made devices and technologies to be translated to market.

Globally, medical device innovators require approval to carry out clinical trials for estimation of the efficacy and safety of their device innovations and progression to market (19). Despite having a population of over 1.3 billion people (20) and carrying 25% of the world disease burden, only 2.5% of clinical trials are conducted in Africa (21). Moreover, few of these trials are locally initiated, and most are sponsored and led by foreign research organizations. The barriers to conducting clinical trials in Sub Saharan African (SSA) countries include complicated logistical systems, inadequate funding, slow regulatory and ethical approval procedures, and poor infrastructure among others (22, 23). The existing inefficiencies in regulatory practices and policies, and lack of infrastructure and facilities lead to high costs for trials, increased participant burden, lengthy and poorly defined clinical trial timelines (24). Furthermore, the weak regulations in SSA raise instances for ethical and safety protocol violations (21).

Despite all these challenges in regulation and clinical evaluation of medical technologies, there are many benefits of conducting trials in SSA such as lower costs, ease of recruiting participants and a diverse population (21). Hence, the number of clinical trials conducted in Africa has more than doubled in the last ten years (25), which has caused various SSA countries to set up enabling environments for research and innovation. For example, Uganda, which has recorded the third highest number of clinical trials in various reviews (25–27) has made efforts to harmonize medical device classification and regulations (1). Controls for the importation and registration of devices have also been instituted by the National Drug Authority of Uganda (9) and capacity has been developed for clinical trials for devices applicable to malaria, HIV and Tuberculosis (2). However, there is still a lot to be done in streamlining and strengthening the regulatory pathway for IMDs in Uganda.

Therefore, this study undertook a mapping of the systems and processes of medical devices innovation and regulation in Uganda, with the aim of evaluating the current knowledge, systems and infrastructure for medical devices regulation and innovation amongst key stakeholders in Uganda.

## 2. Methods

### 2.1. Design

A mixed methods study design using the methods triangulation strategy by combining both quantitative and qualitative data collection approaches was employed in this study (28, 29). Data of equal weight was collected sequentially, first through structured digital questionnaires sent out to innovators followed by a single focus group discussion involving both medical device innovators and regulators. Methods triangulation was used because it provides for more detailed exploration of study variables and reduces bias from either the questionnaire or focus group discussion (30). Individuals, groups (incubation hubs), and communities (research groups) were approached to gain multiple perspectives on the medical device innovation and regulatory environment, to increase data reliability and validity.

### 2.2. Sampling technique

The questionnaire was sent out to respondents selected through snowball sampling (31). The researchers identified some medical device innovators and after explaining the purpose and aims of the study, obtained consent, and asked them to complete the questionnaire. The initial respondents were then asked to forward the questionnaire to other medical device innovators in their circles *via* their email or social media platforms particularly WhatsApp and Twitter which are more commonly used in Uganda (32). The targeted sample size was thirty respondents. This was chosen basing on the methods and tables generated by Louangrath for sample-size estimation for non-finite populations at 95% confidence interval and 5% margin of error (33, 34). All respondents were recruited and completed questionnaires were collected. The researchers terminated the recruitment of participants when no new responses were recorded after four weeks. This method was used because the number of medical device innovators in Uganda has not been published, and the few that are known are scattered around the country.

Participants in the focus group discussion were selected through purposive sampling. Through literature (1, 2, 9) and knowledge of the local regulatory bodies in Uganda, the researchers enlisted key departments in the bodies that participated specifically in medical device regulation. Department heads and representatives were formally invited to the discussion through their supervisors. Innovators who had indicated in their questionnaire responses that they had conducted clinical trials for their medical device innovation were invited for the discussion. The targeted sample size was ten participants. Focus group discussions were chosen to allow for interaction of both regulators and innovators to generate valuable insights in processes and systems under evaluation (35).

### 2.3. Data collection

A structured digital questionnaire was sent out to medical device innovators. A medical device innovator was defined as an individual involved in creating and transforming new ideas and knowledge into new medical devices and technologies intended to improve quality of care, create new and improved diagnostic and treatment protocols, and create patient and service provider networks (36). The digital questionnaires were developed using KoBoToolbox (https://www.kobotoolbox.org/). The survey aimed to establish individual knowledge and experience with the medical device design as well as the regulation and evaluation of clinical trials for medical devices. Respondents were asked about their knowledge of device design, clinical trials, and their experience with the regulation of clinical trials.

A single focus group discussion was conducted to collect data about the current regulatory practices for IMDs in Uganda. Participating regulatory bodies included the National Drug Authority (NDA), the Uganda Registration Services Bureau (URSB), the Uganda National Council for Science and Technology (UNCST), and volunteers from the WHO-Africa Medical Devices Regulators Forum (WHO-AMDRF). The key discussion areas were the current regulations and standards for medical devices, regulatory requirements for designing and conducting clinical trials for medical devices, and the experience of the regulators with regulating medical devices and their clinical trials. The discussion was audio recorded after obtaining signed and verbal consent using an IC recorder. Two observers were present and made brief notes on the discussion and interactions of the participants who answered semi-structured, open-ended questions.

### 2.4. Data analysis

The study collected both qualitative and quantitative data. For this reason, two types of analysis were carried out:

**Quantitative Data analysis:** Raw data was exported to an XLS spreadsheet and cleaned. Primary analysis was done using the KoBoToolbox Excel data analyzer developed by the United Nations Office for the Coordination of Humanitarian Affairs (UNOCHA) (37). Univariate and Bivariate analysis was done with Microsoft Excel to summarize the results using graphs and tables.
**Qualitative Data analysis:** Audio recordings from the focus group discussion were transcribed verbatim and analyzed along with the notes taken, using the content thematic analysis method described by Graneheim and Lundeman (38). By this method, meaningful units were extracted from the transcripts and encoded. Codes were grouped into subcategories based on their similarities and subcategories were combined into categories. Themes were then created from these categories.

### 2.5. Ethical considerations

Ethical review and approval were obtained from the Makerere University School of Biomedical Sciences, Research and Ethics Committee (SBS-REC), and the Uganda National Council for Science and Technology (UNSCT). Informed consent was obtained from each participant before their participation in the focus group discussion, and before responding to the questionnaire. To protect their privacy and ensure confidentiality no personal identifiable data were collected in the tools used. All data were coded and de-identified and were only reported in the aggregate.

## 3. Results

The total number of innovators who responded to the questionnaire was small and not an exhaustive representation of all medical device innovators in Uganda. This is because, even though the snowball sampling method that was followed to get respondents is often used to recruit participants with similar characteristics who are more difficult to locate (39, 40), it is possible that some innovators who were not part of the communities or networks through which the questionnaire was circulated were not involved in the study. However, given that the number of medical device innovators in Uganda has not been published or properly defined, this method was the most suitable for identifying respondents to the questionnaire.

### 3.1. Quantitative results

A total of 47 individuals responded to the questionnaire. The demographic characteristics of these participants, including their highest level of education, field of study and current workplace are shown in Table 1. In the analysis, 33 respondents were considered, while 14 were excluded since they had not been involved in medical device innovations. Of the 33 respondents, the majority had been medical device innovators for either 1–2 years or above 5 years (30% and 27% respectively). 10 respondents had worked on one innovation, 14 on two innovations and 6 had worked on at least 5 innovations.

**Table 1:** Participant demographics.

Demographic	Category	Number	%
Gender	Male	18	38
	Female	29	62
Level of education	UACE	7	15
	Ordinary Diploma	3	6
	Advanced Diploma	3	6
	Bachelors	27	57
	Post Graduate Diploma	1	2
	Masters	6	13
Major field of study	Education	1	2
	Engineering, technology, and applied sciences	43	91
	Medicine	3	6
Current workplace	Academic/research institute	22	47
	Industry	2	4
	Innovation Hubs	2	4
	NGO	3	6
	Self Employed	6	13
	Unemployed	5	11
	Other	7	15
Total Number of Respondents	47	100

**Figure 1:**
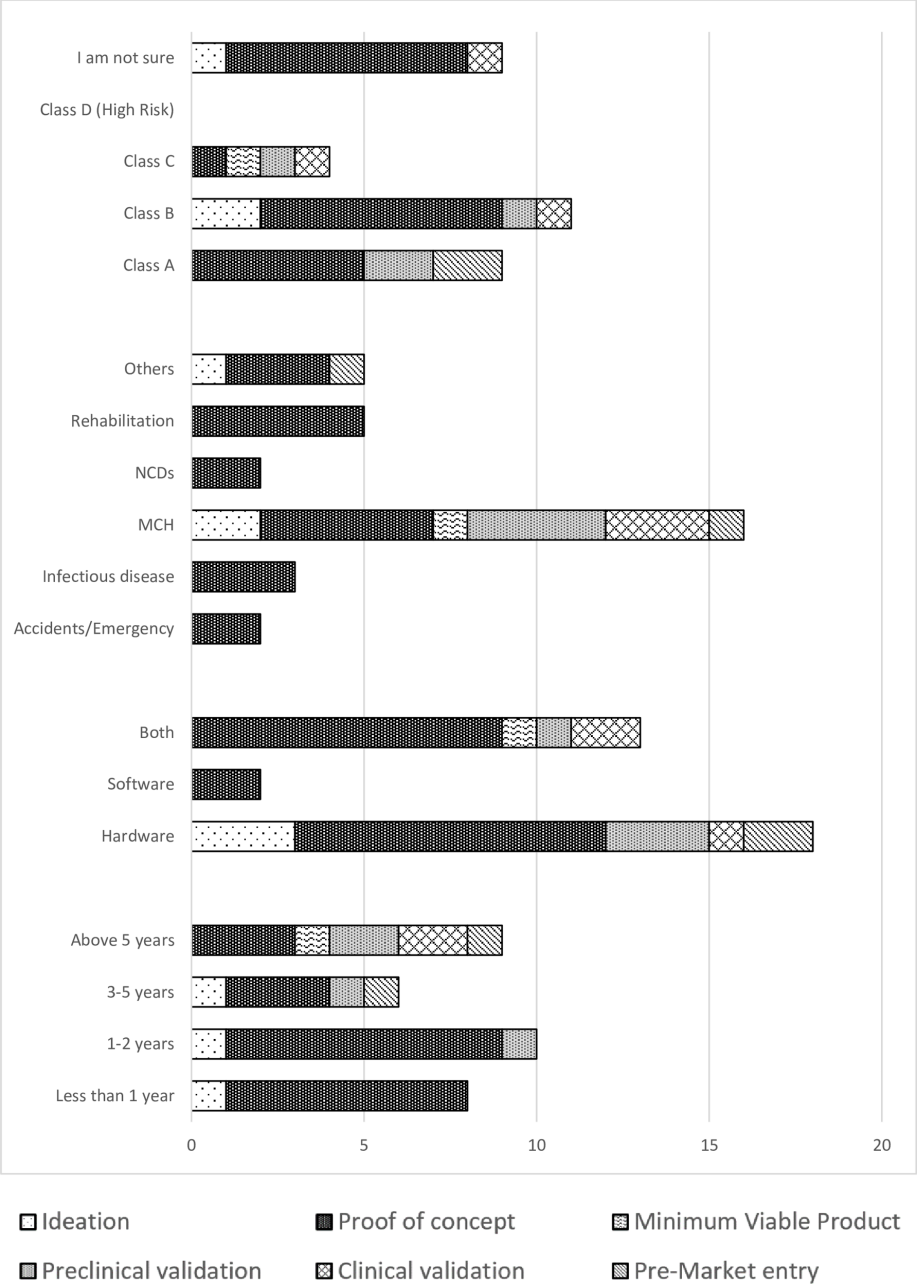
Graph of time spent as an innovator, field of innovation and device classes against the stages of innovation.

**Figure 2:**
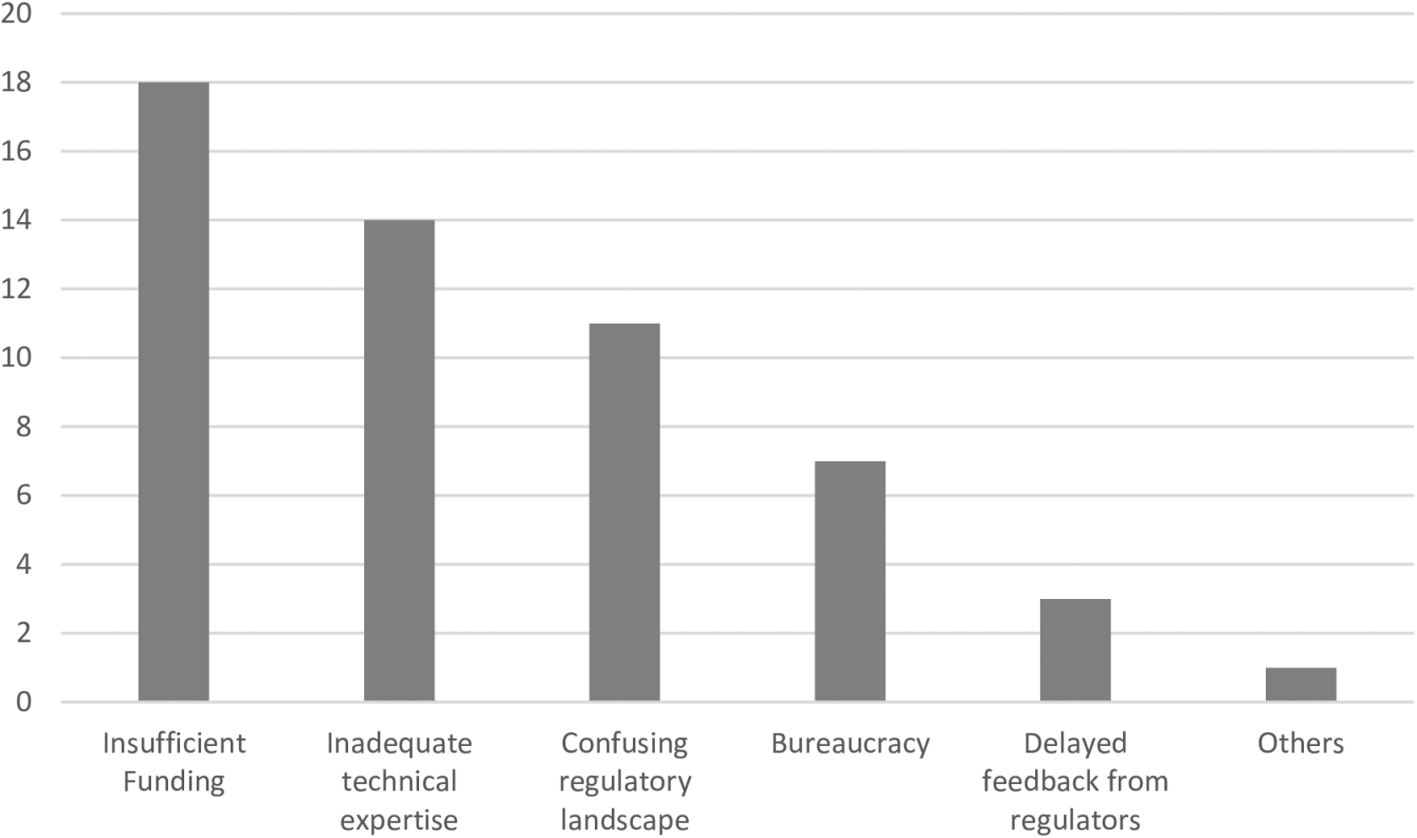
Challenges faced by medical device innovators.

A great majority of the medical device innovators, 48% (*n* = 16) were in maternal and child health (MCH). Others were involved in rehabilitation and assistive technology (*n* = 5), while accidents and emergency and non-communicable diseases had the least number of innovators (*n* = 2). Most of the innovations (*n* = 20) had reached the proof-of-concept stage, while a very small number (*n* = 3) had gone as far as clinical validation. Innovations in the fields of accidents and emergency, rehabilitation and assistive technology, non-communicable diseases and infectious diseases had all just gone as far as the proof-of-concept stage. Majority of innovations were hardware (*n* = 18), both hardware and software (*n* = 13), and only 2 were exclusively software innovations. All software innovations had only gone as far as the prototyping stage (Figure 1). More than half of the respondents (*n* = 22) had stopped working on their innovations, with most of these innovations stalling at the proof-of-concept stage. Insufficient funding, inadequate technical expertise and confusing regulatory landscape were given as the major reason why innovators had stopped working on their innovations (Figure 2). The medical device classification system reported by the NDA was used to classify innovations. Majority of the innovations were reported to belong to class B and class A (*n* = 11 and *n* = 9 respectively), while there were no innovations in class D. Some respondents (*n* = 9) were not sure to which classes their innovation belonged.

Table 2 shows the classification of the innovations in the different fields. 23 respondents thought their innovations required clinical trials (*n* = 5 class A, *n* = 9 class B, *n* = 3 class C). The majority of respondents identified the Uganda National Council of Science and Technology (UNCST) as the body that approves clinical trials in Uganda, while others identified the National Drug Authority, Makerere Clinical Trials unit and others (Table 3). Twenty-seven respondents had never received training in designing clinical trials for medical devices. Those who had received training (*n* = 6) had either attended workshops, researched about, or been mentored in clinical trials for medical devices.

**Table 2:** Class of innovation for medical devices in the various fields of innovation.

	Class A	Class B	Class C	I am not sure
Accidents and Emergency	0	1	0	1
Infectious disease	0	2	1	0
Maternal and Child Health	5	7	3	1
Non- Communicable Diseases	1	1	0	0
Rehabilitation and assistive technology	1	0	0	4
Others	2	0	0	3
Total	9	11	4	9

**Table 3:** Regulators of clinical trials for medical devices.

Institution	No.	%
Uganda National Council for Science and Technology	14	42
Makerere Clinical Trials Unit	6	18
National Drug Authority	6	18
Ministry of Health	3	9
Others	4	13
Total	33	100

### 3.2. Qualitative results

The focus group discussion involved nine participants, four females and five males. Six participants were employed in regulatory institutes in the country and three were active medical device innovators with experience in medical device clinical trials. The different experiences of participants had enabled them to navigate through the medical devices regulatory landscape in Uganda where each played an active role in their capacity. Six categories and two themes emerged from the data on the current regulatory practices for IMDs in Uganda. The two themes were: “developing standards for medical devices regulation” and “Implementation of regulations in practical processes”. Themes and categories are shown in Table 4.

**Table 4:** Themes, categories, and subcategories from the qualitative analysis.

Theme	Category	Subcategory
Developing standards for medical device regulation	Institutional participation in regulation of IMDs	Establishing legal mandate to develop and enforce regulations
		Making use of classification system for medical devices
	Public involvement in developing regulations	Consulting experts in policy review processes
		Establishing demand for standards
	Establishing regional and international collaborations	Adopting international regulatory practices
		Harmonization of standards at regional level
		Subscribing to international regulatory organizations.
Implementation of regulations in practical processes	Establishing a pathway for translation of medical devices	Setting up infrastructure to protect individual ideas
		Approving clinical trials for medical devices
	Providing support for innovators along the process	Providing solutions to straining procedures

#### 3.2.1. Developing standards for medical devices regulation

Participants were actively involved in medical devices regulation as policy makers and standards developers. The theme included three categories: (a) institutional participation in regulation of medical devices, (b) public involvement in developing regulations, and (c) establishing regional and international collaborations for IMD regulation.

Participants identified the National Drug Authority (NDA) as the body that regulates medical devices in Uganda. Conversely, one of the participants mentioned that the NDA does not have the mandate to regulate these devices.

*“There is a limited scope of medical devices that the NDA regulates. Due to legal limitations, NDA does not have the legal mandate to develop medical device standards for locally manufactured products. The current regulation only provides for the control of import and sale of surgical instruments and appliances.”*

Indeed, the role of NDA in the enforcement of the controls of import and sale of devices was noted by the participants.

*“When it comes to the enforcement of (medical device) standards, the NDA have that mandate. I have observed that most of the medical devices are coming from outside Uganda, so there you must get clearance from NDA.”*

The involvement of UNBS in standards development for medical devices has been limited. Public demand was cited as the driving force behind development of any standard. The low demand for medical device standards was given as an explanation for the field having few standards developed:

*“According to the policies and organizational guidelines, UNBS has the mandate to develop and enforce all standards. However, UNBS does not enforce the standards of medical devices. The demand for these (medical device) standards is low. Standards are demand driven. If you do not demand for a standard, UNBS will not develop it. This demand can be individual driven or from a government institution.”*

In the absence of clear regulations and standards, some innovators had opted to follow international regulatory guidelines such as FDA or CE requirements to develop standard medical devices. However, this introduced more challenges for them due to the lack of resources:

*“From the perspective of regulatory strategy, some innovators are choosing to follow CE and FDA regulations. But to get a CE mark and approval, I may not have the capacity or resources and I cannot benefit from this.”*

To solve some of these challenges, the NDA has worked within the confines of the 1993 NDA Act and taken steps to draft and develop regulations for medical devices. NDA has also adopted the medical devices classification system from the International Medical Devices Regulatory Forum. These are applicable only to surgical instruments and appliances. The legal limitations have also prevented them from sharing the classification system with the public:

*“NDA uses Classes A, B, C and D to classify medical devices. That classification was adopted from the international medical devices’ regulators forum. This mode of classification has not been shared with the public because of law constraints, however, it is used internally for the surgical equipment and appliances.”*

Both the NDA and UNBS have also involved experts from the public to develop medical device standards and regulations:

*“NDA has panels of ENT, OBSGYN, General hospital and plastic surgery experts, because normally we want the technical practitioner to give input. Experts from these fields are usually invited to give their opinion about the classification of surgical equipment and appliances.”*

*“UNBS convenes technical committees with different experts across the entire value chain to come up with a working draft of a new standard for approval by the national standards council. We have about 16 indigenous standards that have been developed.”*

The regulatory efforts undertaken by the NDA and UNBS had been noted by the innovators. One innovator noted that although these were commendable, more effort was needed to develop local regulations and standards for medical devices. They noted that developing these standards could take long but adopting and amending them would be more feasible:

*“I understand now, with NDA something can be done. But then the standards must be there, because maybe the regulations help us regulate what is already made but then it might not help facilitate the regulation of what is being developed locally. Many innovators want to see something made in Uganda for Uganda or made for Africa in that regard.”*

*“There is already a code with the IMDRF for medical device regulation to allow developing countries to develop and amend medical device standards. Because, if we focus on developing indigenous standards we might take long.”*

One of the regulators noted that: “the process of developing standards is quite long.” Therefore, the UNBS had in fact decided to adopt standards. In this regard, several standards for medical devices had been adopted from the International Standards Organization (ISO). UNBS has also established regional and international collaborations to ease the development and harmonization of medical device standards. These standards can then be directly applied to the local products in development:

*“We (UNBS) have about 50 standards for medical devices and most of them are ISO adopted. We develop and harmonize standards up to the East African level. In case there is a standard at that level, it governs all the East African countries. There is one standard of medical devices that has successfully been harmonized”*

The WHO and Africa Medical Devices Forum (WHO-AMDF) have also collaborated in harmonization of standards across the continent and have developed guidelines for adoption of standards and regulations for medical devices.

*“The AMDF is developing guidelines that can be adopted by African countries. That is because many of these devices are imported or donated, and some do not match up to what is needed. Countries that are already under the AMDF, for example Uganda, can easily adopt these guidelines and just adjust them a little bit to their specific need.”*

#### 3.2.2. Implementation of regulations in practical processes

All institutions represented in the discussion were involved at a specific stage of the regulatory pathway for IMDs. This theme had two categories: (a) Establishing a pathway translation of medical devices and (b) providing support for innovators along the process. Many innovations are characterized by new ideas and knowledge.

Many innovators are coming up with new innovations and have been encouraged to seek intellectual property (IP) protection. The URSB offers different types of intellectual property (IP) protection and provides opportunity for innovators to apply for IP protection from other countries through continental and international systems.

*“In Uganda, URSB handles intellectual property protection. We offer patents, trademarks, copyright, and design protection. From URSB, you can also make an application to about 20 African countries using the AREPO system. There is also a system under WIPO where you can seek protection in many countries.”*

The regulators also agreed that having IP protection would contribute to translation of an invention to market by increasing visibility of the innovation:

*“If you file for your patent, you are likely to hear from Philips about your innovation. If you do not put your innovation out there, it might die in the lab. You may lack the capacity to scale but if you are looking at making an impact, IP is a means to an end.”*

Another path to translation is through clinical trials. NDA categorizes clinical trials as either *significant* or *non-significant risk*. However, the regulations enacted by the NDA do not include guidelines for medical device clinical trials. As such, they do not have the mandate to regulate these clinical trials:

*“The regulation is silent regarding clinical trials. If you go to NDA seeking approval to do a clinical trial, they tell you they do not have the mandate. In that case they send you to the council for science and technology (UNCST).”*

Another regulator noted this limitation and added that medical device clinical trials for novel devices were referred to the director general for health services:

*“NDA has limitation on this, and since they don”t regulate certain aspects, those cases are referred to the director general of health services in the ministry of health.”*

UNCST has also worked with local research ethics committees (REC) and the NDA to develop a procedure for the approval of clinical trials for novel medical devices:

*“Research Ethics Committees are encouraged to co-opt experts to assess the scientific and clinical aspects of the clinical trial. Those experts do a risk-benefit analysis of the device and then there after they give approval to proceed with the application to UNCST. These cases are then referred to the director general of health services in the ministry of health and then NDA for final approval.”*

An internal evaluation of this process revealed that it was quite long and strenuous. To foster scientific development, a procedure for joint review was developed to reduce the time innovators lose in waiting for approvals:

*“UNCST conducted a study and we found that for a clinical trial to be reviewed and approved in the country it takes about 5 months. Novel medical devices have a mechanism of joint review. The purpose of this mechanism is to enhance the quality of the protocols. It also optimizes the turnaround time of the review. The turn-around time is minimum 32 days depending on the requirements.”*

In fact, one of the innovators had gone through the joint review process and benefitted from the improvements in the approval process:

*“We just completed a clinical trial for our device. We went through the joint review process, though it did not take 32 days like you said but it did not take 6 months either. The hurdles were not so many.”*

**Figure 3:**
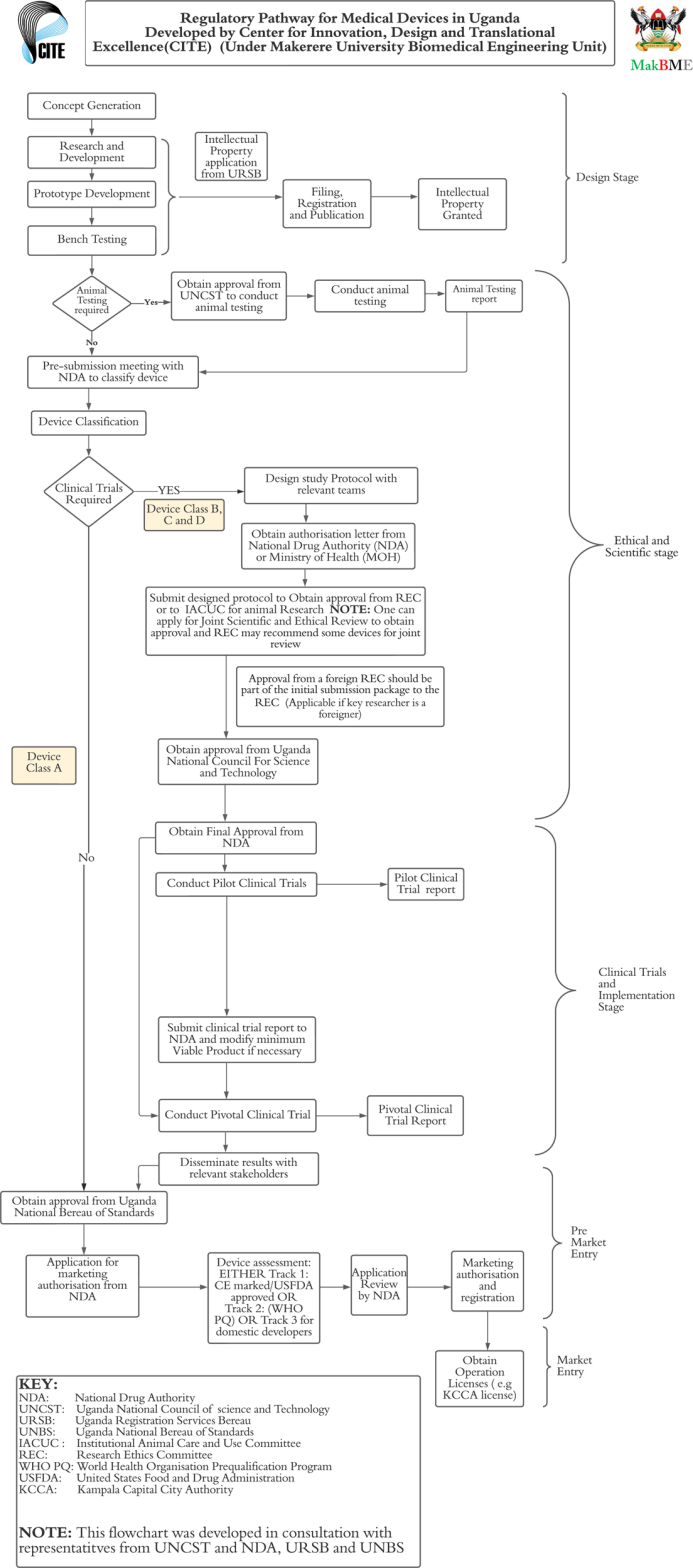
Derived pathway for translation of medical device innovations.

Following the discussion on how these regulations are implemented, participants drew up the pathway shown in Figure 3. This shows various clinical evaluations required for medical devices such as bench and animal testing, pilot, and pivotal studies. IP protection was encouraged for earlier stages of development, specifically after concept generation:

*“The moment there is understanding of the general idea of what you want to do. You may not have everything, but you make an application. You disclose fully what you think it will do, and what you think it cannot do that is possible to physics.”*

The class of device determined whether an innovation required clinical trials or not. Class A devices were considered non-significant risk and did not require clinical trials. Devices in other classes B, C and D were considered significant risk, which required pilot and pivotal clinical trials. This classification has been extended from pharmaceutical products to medical devices:

*“We (NDA) have two kinds of trials for devices. We have significant risk and non-significant risk. Normally the class A devices will fall under the non-significant risk and Class B, C and D will be significant risk.”*

Final approval to manufacture is granted by NDA, after UNBS approval that the device meets all required standards. NDA required manufacturers to comply with ISO 13485, which has been a challenge to innovators.

*The yardstick for inspection for manufacturing is ISO 13485. Of course, most times for local production we fall short. We share the standard with the innovator and ask them to aspire to meet the requirements of that standard. This has been hard because they don't have the resources.”*

## 4. Discussion

This study undertook a mapping of current knowledge, systems and infrastructure for medical devices innovation and regulation in Uganda. The survey established individual knowledge and experience in medical devices innovation and regulation, highlighting challenges and gaps in regulation and translation of medical device innovations. The focus group discussion produced an in-depth review of the regulatory systems and processes for translation of novel medical devices. Two themes of “developing standards for medical devices regulation” and “implementation of regulations in practical processes” emerged from this discussion. This was the first study to document a regulatory pathway for medical devices in Uganda (39, 40).

### 4.1. Medical device innovation landscape in Uganda

Demographic information collected showed that majority of individuals had been innovators for more than a year, held a bachelor's degree with a background in Engineering and applied sciences, and worked in an academic research institute. This demographic is quite prominent of innovators in Uganda (41). These individuals had developed an average of two innovations each, with most of these in MCH. Individuals were mostly involved in developing physical devices that are either solely hardware or systems with both hardware and software components. The growth of innovations has seen Uganda among frontrunners of new approaches to STI due to government, public and private efforts that have been undertaken to foster innovation in Uganda (42).

The government of Uganda like other African governments has been prompted to prioritize STI and invest in high quality higher education to develop human capacity for economic growth and competitiveness (43). This has been done this through programs like the millennium science initiative, an investment of US $33.5 million, which provided training to more than 3,660 scientists and engineers (44), and the Makerere University Research Innovation Fund which has seen total of 775 projects funded through an appropriation of up to 79 billion Uganda Shillings over three financial years since 2019 (45). Such programs have led universities to be the leading source of innovation (46), resulting in more research outputs, especially scientific publications (12).

Universities in Uganda, especially Makerere University, Kyambogo University, and Mbarara University of Science and Technology have also begun admitting and offering an undergraduate program of biomedical engineering designed to equip students with skills in research and innovation of medical devices for diagnosis and treatment of disease to address public health challenges in Uganda (47, 48). This explains the large number of respondents having a bachelor's degree with a background in engineering and applied sciences. The programs offer courses in medical device research, design and innovation as opposed to medicine courses, which also explains why few respondents had a background in medicine. Indeed, graduates of these biomedical engineering programs developed a range of devices that have won grants and international prizes for innovations. For example, the Maternal PPH wrap that seeks to reduce the high mortality around post-partum hemorrhage (49), the EPED Strip a urine-based detection strip for preeclampsia (50), Wekebere a handheld self-diagnostic vital-signs monitoring device for mothers (51) and MScan a portable ultrasound device (52).

Evidently, these innovations and many others like the SAVANT-X baby incubator (53) and the Electronically Controlled Gravity Feed (ECGF) Infusion Set (54) are solving challenges in MCH. This corresponds with the findings that more than half of respondents were involved in innovations in this field. In fact, a need finding study by the Uganda Industrial Research Institute (UIRI) revealed MCH to be the most vulnerable population group in LMICs (55), making it of special interest to innovators. More to that, most public health resources are directed towards MCH conditions, which is one of the three priority areas in sub-Saharan countries (56, 57). In Uganda, government efforts on MCH financing have been through projects such as the Uganda Reproductive, Maternal and Child Health Services Improvement Project (URMCHIP) (58). The government has also worked with donors through voucher programs like the USD 24 million Uganda Voucher plus activity with USAID to increase access and utilization of MCH services (59, 60).

### 4.2. Challenges to medical device innovation and regulation

Most of the innovations in the study had gone as far as the proof-of-concept stage and only three had reached the clinical validation stage, with only one innovator having conducted a clinical trial. In fact, most innovations had stalled at the proof-of-concept stage due to insufficient funding and other challenges. Innovations failing to progress beyond this stage is a common characteristic of innovations in Sub-Saharan Africa where very few new products or technologies, if any, have resulted from the large investments in STI programs (61). This observation has been made by various analyses of innovation systems, such as the global innovation index 2021 which reported that Uganda produced less innovation outputs relative to its level of innovation investments (62). Whereas governments have provided funds towards STI, these funds are usually not allocated to the activities they are intended for (63). Furthermore, financial challenges are often experienced through high interest rates and taxes, numerous transaction costs, and supply chain constraints among others (64).

Besides insufficient funds, confusing regulatory landscape and inadequate technical expertise were other major challenges that led to stalling of innovations. Under the theme of developing standards for medical devices regulations, the confusing regulatory landscape emerged from the absence of a single body fully mandated to regulate medical devices. This makes it difficult to draft, develop and enforce clear regulations and policies for medical devices. Three challenges to the development of medical device standards were shared: legal limitations, lengthy process, and low demand. The legal limitations of the National Drug Policy and Authority Act (65) have prevented the NDA from publicly sharing developed classification systems and expanding their involvement beyond the control of import and sale of surgical instruments and appliances. This is the main reason for the confusing regulatory landscape that was given as a challenge in the survey.

The inadequate technical expertise is indicative of a knowledge gap among innovators. This was also shown by the very few respondents working in industry, and many who could not identify the class of device for their innovation. The lack of channels linking academic institutions to relevant industry greatly limits the expertise innovators are exposed to. In Uganda, knowledge sharing between industry and academia has been limited with poor exposure of industry to the various universities (66). The existing collaborations which would otherwise produce more efficient knowledge flows have limited interactions (42). Other challenges like bureaucracy, delayed feedback from regulators all result from the poorly defined regulatory pathway for medical devices.

### 4.3. Current practices in medical device regulation

The government of Uganda has set up various bodies to regulate medical devices (67). The NDA has taken on a central role in medical device regulation, even though this mandate is not covered under the current law. NDA has fostered collaborations with various institutions like UNBS to develop medical device standards and UNCST to monitor and regulate clinical trials for medical devices as revealed in the FGD. These institutions, especially NDA and UNBS have adopted international standards to address the challenges with the lengthy process of standards development and low demand for medical device standard. Standards have also been harmonized at regional level to ensure an enabling environment for medical device innovation.

The WHO, IMDRF, the International Council for Harmonization of Technical Requirements for Pharmaceuticals for Human Use (ICH) and other international organizations have endeavored to avail guidelines on regulatory processes and offer institutional support to developing countries (68, 69). East African countries including Uganda have subscribed to these organizations to benefit from such programs. Similarly, the Africa Medical Devices Forum (AMDF) issues guidelines for harmonization of standards in Africa (70). Adoption and harmonization of standards has many advantages including: increasing product safety, promoting trade, eliminating the need for redundant testing, fair competition and saving on costs incurred in developing standards locally (71).

In fact, the UNBS has adopted and made available several standards for medical devices such as US ISO 11737–1:2018 for the sterilization of healthcare products, US ISO 15223–1:2016 for medical devices symbols, and various series of ISO 10993 for biological evaluation of medical devices (72). In addition to regional and international collaborations, the public has been involved in regulation of medical devices. The UNBS has a technical committee, UNBS/TC 307, dedicated to medical devices and equipment (72) to review and develop standards for these devices. NDA has expert committees for the review of classification of devices and approval of clinical trials.

Under the second theme of “implementation of regulations in practical processes”, the discussion revealed how the clinical evaluation and regulation of medical devices is currently handled. Following the discussion in Figure 3, the interaction of innovators and various regulatory institutions at different stages of the medical device development process was indicated. Stages of clinical evaluation for medical devices all required approval from NDA and UNCST. This is a technical workaround due to the absence of a single regulatory authority for medical devices. UNCST is mandated to authorize and monitor research in Uganda, while the NDA has extended its regulation of clinical trials to medical devices. Two types of trials were required for medical devices: pilot and pivotal trials. Class A devices are considered non-significant risk by the NDA and are exempt from these trials, while devices in classes B, C and D are considered significant risk and as such undergo clinical trials. These requirements are like those provided by the FDA (4, 5) and the medical device regulations of the EU (6).

To address the overarching challenge of not having a single regulatory authority for medical devices, the Uganda government is working on the National Food and Drugs bill, that seeks to regulate food cosmetics, veterinary products, and medical devices (73). This will streamline the processes for clinical evaluation and regulation of medical devices in Uganda.

## 5. Conclusion

Medical device regulations are required for the innovation, importation, manufacture, and sale of appropriate medical devices. In Uganda, these regulations are not well defined which has led to several challenges for innovation of medical devices. It is still unclear who is responsible for the regulation of locally made medical devices in Uganda. This has resulted in a confusing regulatory landscape for medical devices and has been a great barrier for clinical evaluation and translation of medical devices in the country. Other challenges such as insufficient funds and inadequate technical expertise have also made it difficult to develop innovative medical devices and get them to market. As such, majority of innovations stall at the proof-of-concept stage even with the efforts the government has put to finance STI programs in the country. More to that, legal limitations, lengthy processes, and low demand for standards have also hindered the development of medical device standards and regulations.

Despite these challenges, efforts have been taken to create an enabling environment for medical device innovation and regulation. The NDA has taken on the role of medical device regulation and has worked with UNBS and UNCST to facilitate medical device regulation. Both the NDA and UNBS have established international collaborations to adopt and harmonize standards for medical devices and classification systems. This has seen the NDA adopt the IMDRF medical device classification system and UNBS adopt standards for medical device symbols and biological evaluation of medical devices. These institutions have also encouraged public involvement in developing and enforcing medical device regulations through consultations with expert panels and convening technical committees on standards development.

With these efforts, a pathway has been generated for medical device innovations to be translated to the market. This has enabled some devices to move from the lab and undergo clinical trials even to market, such as the PPH wrap, MScan and Wekebere. The national food and drugs bill that seeks to create a single authority to regulate medical devices will streamline the processes for clinical evaluation and regulation of medical devices in Uganda.

## Data Availability

The raw data supporting the conclusions of this article will be made available by the authors, without undue reservation.
